# Extensive Alternative Splicing of the Repressor Element Silencing Transcription Factor Linked to Cancer

**DOI:** 10.1371/journal.pone.0062217

**Published:** 2013-04-16

**Authors:** Guo-Lin Chen, Gregory M. Miller

**Affiliations:** 1 Division of Neuroscience, New England Primate Research Center, Harvard Medical School, Southborough, Massachusetts, United States of America; International Centre for Genetic Engineering and Biotechnology, Italy

## Abstract

The repressor element silencing transcription factor (REST) is a coordinate transcriptional and epigenetic regulator which functions as a tumor suppressor or an oncogene depending on cellular context, and a truncated splice variant REST4 has been linked to various types of cancer. We performed a comprehensive analysis of alternative splicing (AS) of *REST* by rapid amplification of cDNA ends and PCR amplification of cDNAs from various tissues and cell lines with specific primers. We identified 8 novel alternative exons including an alternate last exon which doubles the *REST* gene boundary, along with numerous 5′/3′ splice sites and ends in the constitutive exons. With the combination of various splicing patterns (e.g. exon skipping and alternative usage of the first and last exons) that are predictive of altered REST activity, at least 45 alternatively spliced variants of coding and non-coding mRNA were expressed in a species- and cell-type/tissue-specific manner with individual differences. By examining the repertoire of *REST* pre-mRNA splicing in 27 patients with kidney, liver and lung cancer, we found that all patients without exception showed differential expression of various *REST* splice variants between paired tumor and adjacent normal tissues, with striking cell-type/tissue and individual differences. Moreover, we revealed that exon 3 skipping, which causes no frame shift but loss of a domain essential for nuclear translocation, was affected by pioglitazone, a highly selective activator of the peroxisome proliferator-activated receptor gamma (PPARγ) which contributes to cell differentiation and tumorigenesis besides its metabolic actions. Accordingly, this study demonstrates an extensive AS of *REST* pre-mRNA which redefines *REST* gene boundary and structure, along with a general but differential link between *REST* pre-mRNA splicing and various types of cancer. These findings advance our understanding of the complex, context-dependent regulation of *REST* gene expression and function, and provide potential biomarkers and therapeutic targets for cancer.

## Introduction

Alternative splicing (AS), a process to differentially link exons in a single precursor mRNA (pre-mRNA) to produce two or more different mature mRNAs, is a major contributor to transcriptome and proteome diversity, with >90% of human genes undergoing AS in a tissue- and developmental stage-specific manner [1], [2]. It is now recognized that the coupling between transcription and splicing is crucial for AS regulation [3], [4], and that exon-intron junctions or splice sites (SS) are specified by epigenetic modifications dependent on cellular context [4], [5]. Accordingly, epigenetic modifications affect not only transcription, but also the co-transcriptional splicing [6], [7]. Epigenetic regulation of pre-mRNA splicing, in line with the spatiotemporal selection of SS, suggest that AS cross-talks with environmental cues to contribute to adaptive responses and disease pathophysiology. Indeed, AS is modulated by the circadian clock, psychological stress, and numerous hormones and chemicals [8]–[10], while an estimated 15–60% of human genetic diseases, ranging from neurological to tumorigenic and metabolic disorders, involve splicing mutations [2], [11]–[14].

The repressor element silencing transcription factor (REST, also known as NRSF for neuron-restrictive silencing factor), originally identified as a repressor of neuronal genes in non-neuronal cells [15], [16], is now recognized as a coordinate transcriptional and epigenetic regulator that orchestrates the cellular epigenome in both neuronal and non-neuronal cells [17]. REST binds to widely distributed genomic regulatory sequences including the repressor element-1 (RE1) by a DNA-binding domain (DBD) which comprises 8 zinc finger motifs (ZFMs), while its effect on gene expression is mediated by two independent repression domains (RD1 and RD2) which directly or indirectly recruit numerous transcriptional and epigenetic cofactors. Briefly, the N-terminal RD1 recruits mSin3, a scaffold for histone deacetylases (HDACs), while the C-terminal RD2 partners with the REST corepressor (CoREST) which additionally recruits HDACs, methyl-CpG binding protein 2 (MeCP2), histone H3K4 lysine demethylase (LSD1) and H3K9 methyltransferases (G9a), as well as a component of the SWI/SNF chromatin remodeling complex-Brg1. By recruiting numerous cofactors to target gene loci, REST promotes dynamic, context-dependent chromatin organization and repression/activation of thousands of genes involved in many cellular processes including tumorigenesis, for which it functions as a tumor suppressor or an oncogene depending on cellular context [18], [19]. The diverse, context-dependent function of REST is specified by multiple mechanisms including proteasomal degradation, nuclear translocation and pre-mRNA splicing [20]–[23], as well as the modulation by non-coding RNAs (ncRNAs) and binding affinity of REST to diverse RE1 and non-RE1 sites [24], [25].

*REST* undergoes AS with a limited number of splice variants having been reported, of which a C-terminal truncated variant REST4, which contains RD1 and ZFMs 1–5, has been well documented [20], [21]. As a dominant negative, REST4 is linked to small cell lung cancer (SCLC), neuroblastoma and breast cancer [26]–[28], and it contributes to early-life programming of the stress response, neuroprotection and hormonal regulation of glutamine synthethase [29]–[31]. Another two splice variants, REST1 which contains RD1 and ZFMs 1–4 [16], and REST-5FΔ with a deletion of the ZFM-5 [27], have also been documented. Notably, REST4 with ZFM-5 is transported to the nucleus while REST1 without ZFM-5 is not [32], and it was later demonstrated that ZFM-5 is essential for the nuclear targeting of REST [33].

In this study, we performed a comprehensive analysis of the AS of *REST* pre-mRNA and examined its relevance to cancer. We demonstrate that: 1) *REST* undergoes extensive AS across a gene boundary now doubled by a novel last exon (E_5_), with numerous coding and non-coding mRNAs being formed with a species- and cell-type/tissue-specific expression; 2) numerous *REST* splice variants, which are caused by various splicing patterns (e.g. exon skipping and alternative usage of the first and last exons) predictive of altered REST activity, are generally but differentially linked to various types of cancer; and 3) exon 3 (E_3_) skipping, which causes no frame shift but loss of ZFM-5 essential for nuclear translocation, is remarkably affected by pioglitazone, a highly selective agonist for PPARγ which modulates cell differentiation and tumorigenesis besides its metabolic actions. These findings advance our understanding of the complexity of *REST* gene regulation and function, and provide potential biomarkers and therapeutic targets for cancer.

## Materials and Methods

### Ethics Statement

The use of human tissues was approved by the Harvard Institutional Review Board, and the related projects for which macaques and mice were euthanized were approved by the Institutional Animal Care and Use Committee for Harvard Medical School. The Harvard Medical School animal management program is accredited by the American Association for the Accreditation of Laboratory Animal Care (AAALAC) and meets National Institutes of Health standards as set forth in the Guide for the Care and Use of Laboratory Animals (DHHS Publication No. (NIH) 85–23 Revised 1985). The institution also accepts as mandatory the PHS Policy on Humane Care and Use of Laboratory Animals by Awardee Institutions and NIH Principles for the Utilization and Care of Vertebrate Animals Used in Testing, Research, and Training.

Animals (macaques and mice) involved in this study were cared for in compliance with National Institutes of Health, US Department of Agriculture, and Harvard Medical School guidelines for animal research. Macaques were single-housed and all efforts were made to reduce discomfort and provide enrichment opportunities (e.g. varied food supplements, foraging and task-oriented feeding methods, and interaction with caregivers and research staff). Specifically, macaques were fed twice daily (AM and PM) with a balanced commercially available Old World Primate Diet (e.g. Harlan Teklad 8714 Monkey Diet). Fruit and/or vegetable supplements were provided to all animals daily, and drinking water was provided ad libitum by automatic water lixits or plastic water bottles. Tissue samples were collected from five macaques which had been utilized in other experiments at the time of their necropsy. The macaques were euthanized following being anesthetized with ketamine HCl by an intravenous pentobarbital overdose, and exsanguinated. Mice were euthanized with carbon dioxide gas inhalation followed by cervical dislocation, and all efforts were made to minimize suffering.

### Human and animal tissues

The cDNA samples derived from adult normal human tissues (kidney, liver, lung, pituitary, hippocampus, amygdala and pons, 1 for each) were purchased from the BioChain® Institute, Inc (Newark, CA). 27 pairs of tumor and adjacent normal tissues from patients diagnosed clinically with kidney, liver and lung cancers (9 pairs for each) were obtained from the UMass Cancer Center Tissue Bank (5 pairs for each cancer as tissue in RNAlater) and the BioChain® Institute Inc (4 pairs for each cancer as total RNA). The demographic information of the patients briefly shown in Table 1. The human peripheral blood mononuclear cells (PBMCs), which were purified as described previously [34], were kindly gifted by Dr. Fred Wang at the Brigham & Women's Hospital. We also collected tissues from rhesus monkeys and mice euthanized for other projects.

**Table 1:** Demographic data for the patients with cancer.

ID#	Gender	Age(y)	Basic Diagnosis	Grade
Kidney cancer				
Ki#1	Male	2	Nephroblastoma	n.a.
Ki#2	Male	45	Renal Cell Carcinoma	2
Ki#3	Male	43	Renal Cell Carcinoma	2
Ki#4	Female	50	Renal Cell Carcinoma	2
Ki#5	Male	52	Renal Cell Carcinoma	2
Ki#6	Female	75	Renal Cell Carcinoma	2
Ki#7	Male	53	Renal Cell Carcinoma	n.a.
Ki#8	Male	63	Renal Cell Carcinoma	n.a.
Ki#9	Female	55	Adenocarcinoma	n.a.
Liver cancer				
Li#1	Male	59	Hepatocellular Carcinoma	3
Li#2	Male	59	Hepatocellular Carcinoma	n.a.
Li#3	Female	71	Hepatocellular Carcinoma	3
Li#4	Male	58	Cholangiocarcinoma	2
Li#5	Male	59	Cholangiocarcinoma	2
Li#6	Female	36	Cholangiocarcinoma	2
Li#7	Male	60	Hepatocellular Carcinoma	n.a.
Li#8	Male	33	Hepatocellular Carcinoma	n.a.
Li#9	Male	47	Hepatocellular Carcinoma	n.a.
Lung cancer				
Lu#1	Male	51	Adenocarcinoma	3
Lu#2	Male	63	Squamous Cell Carcinoma	n.a.
Lu#3	Male	64	Adenocarcinoma	3
Lu#4	Male	51	Adenocarcinoma	3
Lu#5	Male	61	Adenocarcinoma	3
Lu#6	Male	63	Adenocarcinoma (100%)	3
Lu#7	Male	46	Squamous cell carcinoma	n.a.
Lu#8	Male	63	Squamous cell carcinoma	n.a.
Lu#9	Male	67	Squamous cell carcinoma	n.a.

Note: The grade information is not available for samples from BioChain^®^ Institute, Inc.

### Cell lines and drug treatment

A total of 18 cell lines derived from human (HEK293, HEK293T, HepG2, NCCIT, SH-SY5Y, A549, MCF7, K562, SK-N-MC, HeLa, Raji, TE671, Jurkat, Sup-T1 and induced pluripotent stem (iPS)), nonhuman primate (COS-7) and rodents (RN46A and PC12) were employed in this study. Except for RN46A and iPS, all the other 16 cell lines were obtained from American Type Culture Collection (Manassas, VA). RN46A cells were kindly provided by Dr. Scott Whittemore [35], while the iPS cells were originated from Dr. Stephen J. Haggarty [36]. To examine the effect of pioglitazone on *REST* pre-mRNA splicing, the NCCIT, HEK293T and HepG2 cells were treated with either 10 µM of pioglitazone (Sigma-Aldrich) or a matched concentration of the solvent (0.04% DMSO), and cells were harvested at 48 hours following treatment. Treatments were performed in duplicate on 3 independent occasions.

### RNA isolation and cDNA synthesis

Total RNA was extracted using Trizol® reagent (Invitrogen). An aliquot of total RNA was reverse transcribed into cDNA using the QuantiTect® Reverse Transcription Kit (Qiagen), while another aliquot was reverse transcribed into cDNA by using an anchored oligo-dT (anchor sequence given in Table 2). Synthesized cDNA was diluted to 50 ng/µl for use.

**Table 2:** Oligos used for the detection of specific *REST* splice variants.

Name	Sequence	Note
*Standard/nested PCR*		
*E_1a_F_1_	5′-cgaaactccagcaacaaaga-3′	
E_1a_F_2_	5′-ccagcacccaactttaccac-3′	
*E_1b_F_1_	5′-agaagcccggacgccggct-3′	
E_1b_F_2_	5′-tcggagaagcccggacgc-3′	
*E_1c_F_1_	5′-gatggcatttgcttccaact-3′	
E_1c_F_2_	5′-cctggacggtcttcctaaca-3′	
E_2_F_1_	5′-cagtgagcgagtatcactgga-3′	
E_4_R_1_	5′-ctgcactgatcacatttaaatg-3′	
E_4_R_2_	5'-caaactaagaactgaaaccttgttca-3′	
E_4_R_3_	5′-cacataactgcactgatcacattta-3′	
E_5_R_1_	5′-ccctgtgcatatcacctcct-3′	
E_5_R_2_	5′-agggaaatcagtcagcttgg-3′	
rhesus-E_1a_F_2_	5′-ccagcacccaacttttccac-3′	
rhesus-E_4_R_3_	5′-cacgtaactgcactgatcacattta-3′	
mouse-E_1a_F_2_	5′-cctcgacgcccaacttttcc-3′	
mouse-E_4_R_3_	5′-cacataattgcactgatcacattta-3′	
rat-E_2_F_1_	5′-cagtcagcgaataccactggc-3′	
rat-E_5_R_1_	5′-tgtggcttccaacttccttc-3′	
rat-E_5_R_2_	5′-agggaaatcaatcagcctgg-3′	
*E_2_R_1_	5′-tggaaaggtcatgcaagtca-3′	
E_2_R_2_	5′-atggaacctggatttgaacc-3′	
Oligo-dT anchor	5′-gaccacgcgtatcgatgtcgac-3′	
*qRT-PCR*		
E_1a_/E_3_-F	5′-cgaggaaggccggagaac-3′	SYBR Green I
E_1a_/E_3_-R	5′-gcccattgtgaacctgtctt-3′	
E_1a_/E_4_-F	5′-gaggaaggccggtgagaa-3′	SYBR Green I
E_1a_/E_4_-R	5′-cgtgggttcacatgtagctct-3′	
E_1a_/E_2_-#43F	5′-cgaggaaggccgaataca-3′	Hybridization Probe
E_1a_/E_2_-#43R	5′-agccctcctcctccagaa-3′	
E_1c_/E_2_-#43F	5′-tctgtgatggcatttgcttc-3′	Hybridization Probe
E_1c_/E_2_-#43R	5′-gttgccactgctggtaaaca-3′	
E_3_/E_5_-#88F	5′-gacatatgcgtactcattcagagc-3′	Hybridization Probe
E_3_/E_5_-#88R	5′-ttggtttacagtcgtggcttc-3′	
E_2_-F_1_	5′-accgaccaggtaatcacagc-3′	SYBR Green I
E_2_-R_1_	5′-ctggtgtggtgtttcaggtg-3′	
GAPDH-F_1_	5′-tgccctcaacgaccactttg-3′	SYBR Green I
GAPDH-R_1_	5′-tctctcttcctcttgtgctcttgc-3′	

* The E_1a_/E_2_, E_1b_/E_2_, E_1c_/E_2_ junctions can be quantified by SYBR Green I qRT-PCR using the E_1a_F_1_, E_1b_F_1_ and E_1c_F_1_ paired with E_2_R_1_, respectively.

### PCR amplification and DNA sequencing

A touchdown PCR protocol was employed for both standard and nested PCRs, and amplifications were performed in a MJ Research PTC-200 Peltier Thermal Cycler (GMI) in a total volume of 20 µl comprising 1 µl of template (50 ng/µl cDNA for standard or 1^st^ step PCR, and 1∶20 diluted product of 1^st^ step PCR for 2^nd^ step of nested PCR), 10 pmoles of each primer (Table 2), and 10 µl of GoTaq® Green Master Mix (Promega). The first step of nested PCR was performed by using cDNA made by anchored oligo-dT as template and oligo-dT anchor paired with E_1a_F_2_, E_1b_F_2_ and E_1c_F_2_ as the primer sets. For adult normal human tissues without cDNA made by anchored oligo-dT available, the oligo-dT anchor was replaced by E_4_R_3_ and E_5_R_2_ (outside E_4_R_1_ and E_5_R_1_, respectively). Primers for rhesus macaque and rodents were modified if necessary. Amplification conditions involved an initial 2.5 min denaturation at 95°C, followed by 28 (for 1^st^ step of nested PCR) or 40 (for standard or 2^nd^ step of nested PCR) cycles of 30 s denaturation at 95°C, 30 s annealing (temperature starting at 61°C and decreased by 0.5°C/cycle for the initial 12 cycles, then fixed at 55°C), and 60∼180 s extension at 72°C, with a final extension of 5 min at 72°C. PCR products were loaded on a 2% agarose gel and amplicons of distinct size were excised, purified and sequenced. DNA sequencing was performed as commercial service by the Functional Biosciences Inc (Madison, WI). PCR products with poor quality sequencing data were cloned into pGEM®-T vector (Promega) for further sequencing.

### 5′/3′ Rapid amplification of cDNA ends (RACE)

Total RNA generated from HEK293, HEK293T, HepG2 and SH-SY5Y were used to perform the 5′ and 3′ RACE, which were carried out by two commercial kits, the 5′/3′ RACE Kit (2^nd^ Generation) from Roche (Indianapolis, IN) and GeneRacer® from Invitrogen, which differ in strategies for 5′ RACE. For 5′ RACE with GeneRacer® kit, a RNA Oligo was firstly ligated to RNA, followed by cDNA synthesis using Oligo-dT and nested PCR using *REST*-specific reverse primers (e.g. E_4_R_1_ and E_4_R_2_) paired with a GeneRacer® 5′ primer (homologous to the RNA Oligo). For 5′ RACE with Roche's kit, cDNA was first synthesized from total RNA using a *REST*-specific reverse primer (e.g. E_4_R_2_, outside), followed by tailing with dATP and terminale transferase and subsequent nested PCR using anchored Oligo-dT or anchor primer paired with a *REST*-specific reverse primer (e.g. E_4_R_1_, inside). To perform 3′ RACE, cDNA was synthesized from total RNA using anchored Oligo-dT, followed by nested PCR using *REST*-specific forward primers (e.g. E_1a_F_1_ and E_1a_F_2_) paired with the anchor primer.

### Quantitative real-time PCR (qRT-PCR) assay

Using primers listed in Table 2, we developed SYBR Green I and/or Hybridization Probe qRT-PCR for specific exon-exon junctions and exon 2 (E_2_, presumably represents overall expression of coding mRNAs regardless of the first and last exons), as well as the housekeeping gene *GAPDH*. The qRT-PCR assays were performed as previously described on a Roche LightCycler 2.0 system [37], with the threshold cycle (Ct) values being employed to evaluate the expression change between paired tissues or cells by the 2^−ΔΔCt^ approach [38]. PCR reactions were run in duplicate. For E_1a_/E_4_ junction which is expressed at low levels in most cases, only SYBR Green I assay is available and the Ct values are likely confounded by primer dimer formed by excessive remaining primers, so we performed qRT-PCR by using the product of the 1^st^ step PCR as template. Owing to the high sequence identity between the 3′ ends of E_2_ and E_3_, qRT-PCR assay specific for E_2_/E_4_ junction was not attainable.

### Bioinformatics and data analysis

Prediction of the open reading frame (ORF) of specific *REST* variants was performed by using the StarORF program (http://star.mit.edu/orf/runapp.html), and epigenetic information at the *REST* locus was retrieved from the UCSC Genome Browser (http://genome.ucsc.edu/cgi-bin/hgGateway). Comparisons of qRT-PCR-assayed expression levels of specific exon-exon junctions between paired tissues or cells were carried out by the 2^−ΔΔCt^ approach using appropriate reference, and two-fold change was considered as significant.

## Results

### Identification of E_2_/E_3_ skipping expressed in a species-dependent manner

**Figure 1:**
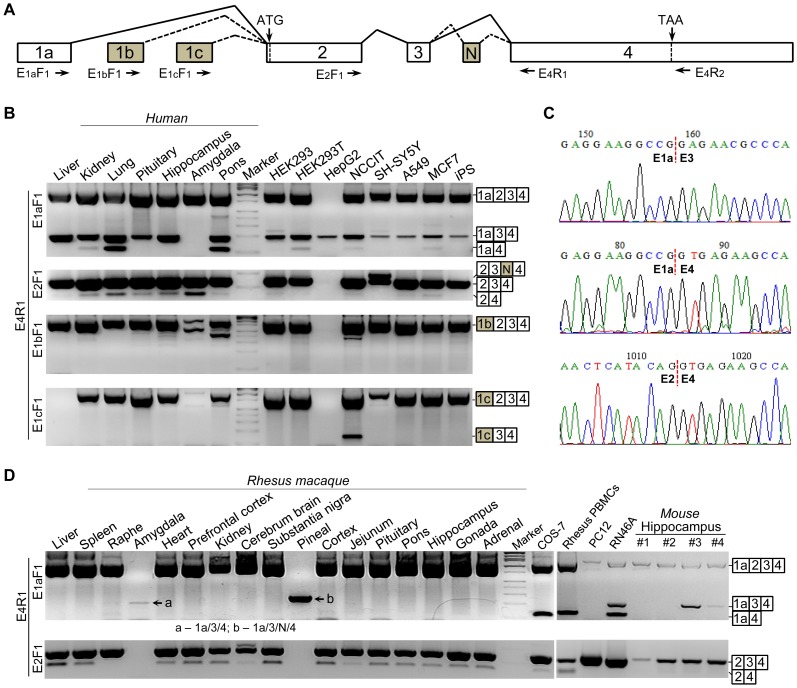
Schematic structure of *REST* gene and identification of E_2_/E_3_ skipping. (**A**) Illustration of annotated *REST* exons and locations of primers employed for the identification of *REST* splice variants. The constitutive exons (1a, 2, 3 and 4) and alternative exons (1b, 1c and N) are shown in open and gray boxes, respectively, while the forward and reverse primers are indicated by right and left arrows, respectively. (**B**) Detection of E_2_/E_3_ skipping in human tissues and cell lines by nested PCRs with specific primer sets. E_2_/E_3_ skipping is commonly associated with E_1a_ but rarely associated with E_1b_ and E_1c_. (**C**) Trace chromatograms for exon-exon junctions involved in E_2_/E_3_ skipping. (**D**) Detection of E_2_/E_3_ skipping in tissues and cell lines from nonhuman primates and rodents by nested PCR. Of the numerous rhesus tissues, E_2_ skipping alone was only observed in amygdale (a) and pineal (b).

We performed standard and nested PCRs with cDNA samples derived from numerous human tissues (liver, kidney, lung, pituitary, hippocampus, amygdala and pons) and cell lines (HEK293, HEK293T, HepG2, NCCIT, SH-SY5Y, A549, MCF7 and iPS). Using a reverse primer E_4_R_1_ targeting the proximal exon 4 (E_4_) paired with the forward primers E_1a_F_1_ and E_2_F_1_ targeting exons 1a (E_1a_) and 2 (E_2_) (Figure 1A and Table 2), respectively, we identified several *REST* splice variants with E_2_ and/or E_3_ skipped (Figure 1B and 1C). The variant with E_2_ skipped is abundantly expressed in all tested cell lines and tissues except amygdala, while the variants with E_3_ alone or plus E_2_ skipped were expressed at low levels in a subset of tissues and cell lines. Notably, E_2_/E_3_ skipping is predominantly associated with E_1a_ but rarely with E_1b_ and E_1c_ (Figure 1B). In addition, E_2_/E_3_ skipping was observed in another 7 human cell lines and the peripheral blood mononuclear cells (PBMCs) (Figure S1).

We tested whether E_2_/E_3_ skipping is expressed in nonhuman primate and rodent tissues and cell lines. As shown in Figure 1D, skipping of E_2_ alone was only observed in amygdala and pineal out of 17 tissues obtained from 1 rhesus macaque, while skipping of both E_2_ and E_3_ was only observed in macaque PBMCs and COS-7 cells (derived from African green monkey kidney); however, skipping of E_3_ alone was observed in most macaque tissues and COS-7 cell line. In rodents, skipping of E_2_ (alone and plus E_3_) was observed in RN46A but not PC12 cells, while skipping of E_2_ alone was observed in the hippocampus from 2 of 4 mice tested, suggesting an inter-individual difference. Similarly, individual difference in *REST* pre-mRNA splicing was also observed in the pons and raphe from 4 macaques (Figure S2).

### Discovery of a novel last exon which doubles human *REST* gene boundary

**Figure 2:**
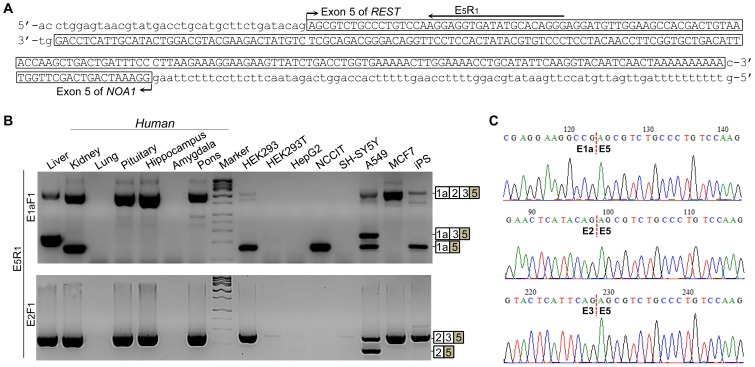
Identification of a novel last exon (E_5_) of *REST*. (**A**) Sequence of E_5_ and its partial (81-bp) overlapping with *NOA1* gene in opposite direction. (**B**) Detection of E_5_ inclusion in human tissues and cell lines by nested PCR. (**C**) Trace chromatograms for junctions of E_5_ with E_1a_, E_2_ and E_3_. Note that the 3′ ends of E_2_ and E_3_ share high sequence identity.

**Figure 3:**
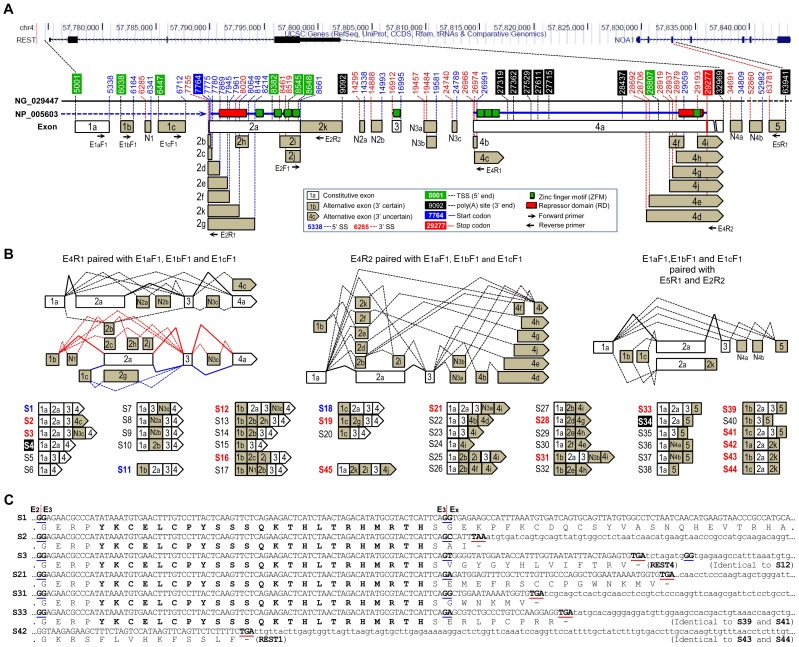
Details of the identified *REST* exons, splicing patterns and resultant splice variants. (**A**) Identified *REST* exons and their 5′/3′ SS or ends. The UCSC genes track was used to briefly indicate the chromosomal location of *REST* gene locus, while the genomic sequence NG_029447 was employed as a reference to clarify the exact positions of the exons. Major domains of the REST protein (NP_005603) were illustrated parallel to the corresponding coding region so as to predict consequences of the AS of *REST* pre-mRNA at the protein level. The constitutive and alternative exons, as well as their 5′/3′ SS or ends, are color-coded as indicated. Note that: 1) exon N in Figure 1 is renamed as N_3c_ here; 2) E_2k_ is an extension of E_2a_ without splicing; and 3) E_5_ and N4b are not actually presented in NG_029447. (**B**) Patterns of *REST* pre-mRNA splicing and resultant mRNA variants. Putative coding variants are color-coded (blue–no amino acid change predicted; red-truncated protein) or shadowed (loss of ZFM-5). (**C**) Prediction of C-terminal truncated REST proteins for specific mRNA variants. The exon-exon junctions and premature stop codons are underlined.

We performed RACE to determine the 5′ and 3′ ends of human *REST* mRNA. Unexpectedly, we identified a novel polyadenylated exon (E_5_) that locates ∼30 kb downstream of E_4_ and partially overlaps in opposite direction with exon 5 of the nitric oxide associated-1 (*NOA1*) gene (Figure 2A), which encodes a GTPase essential for mitochondrial protein synthesis [39]. By nested PCRs using an E_5_-specific reverse primer (E_5_R_1_) paired with E_1a_F_1_ and E_2_F_1_, respectively, we found that E_5_ inclusion, occasionally in combination with E_2_/E_3_ skipping, is expressed in most human tissues and cell lines (Figure 2B, 2C and Figure S1). Like E_2_/E_3_ skipping, E_5_ inclusion is primarily associated with E_1a_ but rarely with E_1b_ and E_1c_. Notably, we found no variants containing both E_4_ and E_5_, suggesting that E_4_ and E_5_ are mutually exclusive and that E_4_, like the case for E_2_ and E_3_, can be completely skipped. Accordingly, the novel last exon E_5_ doubles the human *REST* gene boundary from ∼28 kb to ∼59 kb (Figure 3A). E_5_ inclusion was not observed in nonhuman primates and rodents.

### Extensive AS of *REST* pre-mRNA

By examining above-mentioned primate tissues and cell lines, as well as paired tumor and adjacent normal tissues from cancer patients mentioned hereinafter, we revealed an extensive AS of *REST* pre-mRNA. As shown in Figure 3A, besides E_5_ and the previously reported exon N (now renamed as N_3c_), we identified another 7 novel alternative exons (N_1_, N_2a_, N_2b_, N_3a_, N_3b_, N_4a_ and N_4b_), along with numerous 5′/3′ SS and ends in the constitutive exons E_2_ and E_4_. Regardless of the 5′/3′ ends in E_2_ and E_4_, at least 45 variants (S1–S45) are formed by various splicing patterns including exon skipping, alternative 5′/3′ SS, mutual exclusion and alternative usage of the first and last exons (Figure 3B). Sequences of the novel variants have been deposited in GenBank with assigned accession numbers JX896957-JX896993 and KC117262-KC117266.

29 of the 45 splice variants involve full or partial skipping of E_2_ where translation initiates, such that they may function as ncRNAs due to lack of the translation start site (TSS), except that several variants (S16, S19 and S28) whose TSS was preserved are predictive of N-terminal truncated REST protein isoforms. Similarly, partial or complete skipping of E_4_, which is frequently accompanied by E_2_ skipping and/or E_5_ inclusion, produces numerous ncRNAs or coding mRNAs predictive of truncated REST protein isoforms. In contrast, skipping of the 84-bp E_3_ (S4 and S34) causes no frame shift but loss of ZFM-5. Notably, variants with intact E_2_ (or plus E_3_) followed by alternative exons (but not E_4_) are predicted to encode C-terminal truncated REST proteins, of which 8 variants with E_3_ encode REST4 (S3 and S12) or REST4-like (S2, S21, S31, S33, S39 and S41) proteins with RD1 and ZFMs 1–5, while another 4 variants (S34, S39–S41) with E_3_ skipped encode REST1 with RD1 and ZFMs 1–4 (Figure 3C).

Most of the 45 splice variants are expressed at low levels in a cell-type/tissue-specific manner; however, at least one variant was observed in all the tested tissues and cell lines (Figure 1, 2 and Figure S3).

### Link between *REST* pre-mRNA splicing and cancer

**Figure 4:**
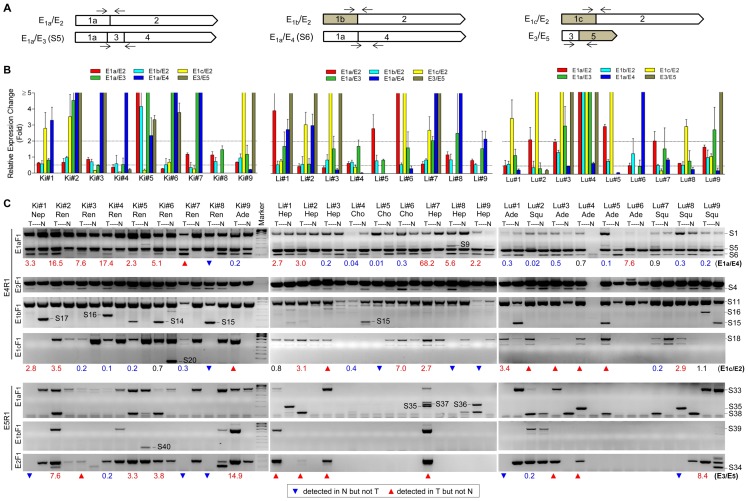
Comparison of *REST* pre-mRNA splicing profile between paired tumor (T) and adjacent normal (N) tissues. (**A**) Strategy for the design of qRT-PCR assay for specific exon-exon junctions. (**B**) Expression changes (T over N, in fold) of specific exon-exon junctions assayed by qRT-PCR assay; (**C**) Expression of specific *REST* splice variants detected by nested PCR. The forward and reverse primers are indicated by right and left arrows, respectively. Paired T and N tissues were collected from 27 patients with kidney (Ki), liver (Li) and lung (Lu), and the basic diagnosis shown in Table 1 was briefly given as its initial 3 letters for each patient. Expression changes (T over N, in fold) of specific variants or splicing (e.g. exon-exon junctions) were assayed by qRT-PCR and calculated by the 2^−ΔΔCt^ approach using E_2_ as the reference. Data are shown as Mean±SEM. qRT-PCR assay is not attainable for E_3_ skipping only (i.e. E_2_/E_4_ junction), but apparent changes can be observed between T and N tissues for most subjects by nested PCR using the E_2_F_1_/E_4_R_1_ primer set.

We compared the expression of specific splicing patterns and variants between paired tumor (T) and adjacent normal (N) tissues from 27 patients with kidney (Ki), liver (Li) and lung (Lu) cancers (9 pairs for each cancer, Table 1) by nested PCR and qRT-PCR assays, with a focus on E_2_/E_3_ skipping and alternative usage of the first and last exons. The qRT-PCR assays were designed targeting specific exon-exon junctions (Figure 4A), and expression changes of specific splicing between T and N are shown in folds (T over N) for each subject (Figure 4B). With the exception of E_1a_/E_3_ and E_1a_/E_4_ which represent S5 and S6 variants, respectively, the exon-exon junctions do not necessarily represent a single specific splice variant, which however can be detected by nested PCRs (Figure 4C). Accordingly, both qRT-PCR and nested PCR assays were taken into consideration for the comparison of *REST* pre-mRNA splicing profile between paired T and N tissues. While the wide expression of E_2_/E_3_ skipping and E_5_ inclusion in human was further validated, we found that all the 27 patients without exception showed differential expression of numerous *REST* splice variants caused by specific splicing patterns between paired T and N tissues, with a striking tissue-type and individual difference.

As shown in Figure 4B and 4C, variants with E_2_/E_3_ skipped were differentially expressed between paired T and N tissues for most patients. Of variants using E_4_ as the last exon, S6 (both E_2_ and E_3_ skipped) showed strikingly differential expression for all the patients except Lu#4 and 7. Specifically, 7, 5 and 1 patients with kidney, liver and lung cancer, respectively, showed increased S6 expression, whereas 2, 4 and 6 patients with kidney, liver and lung cancer, respectively, exhibited decreased S6 expression. Although qRT-PCR assay for E_3_ skipping only (i.e. E_2_/E_4_ junction) is not attainable, nested PCR with E_2_F_1_/E_4_R_1_ showed apparently differential expression of S4 between paired T and N for a portion of patients. Meanwhile, differential expression of E_2_-skipped variants S5 and S15 between paired T and N from some patients was shown by qRT-PCR and nested PCR, respectively. Moreover, some other variants (e.g. S14, S16 and S17) with E_1b_ as the first exon and E_2_ partially skipped were differentially expressed between paired T and N for a few patients. For example, S14 and S16 were only observed in the T tissues of 4 patients (Ki#5, 6 for S14 and Ki#4, Lu#9 for S16, respectively). Likewise, of variants using E_5_ as the last exon, S34 (E_3_ skipped), S35 (E_2_ skipped) and S38 (both E_2_ and E_3_ skipped) showed apparently differential expression between paired T and N as indicated by nested PCRs with E_1a_F_1_/E_5_R_1_ and E_2_F_1_/E_5_R_1_. Accordingly, E_2_/E_3_ (especially E_3_) skipping is generally but differentially linked to different types of cancer with striking cell-type/tissue and individual differences.

As shown in Figure 4C, with the exception of 3 patients (Li#5, 6 and Lu#6) without detectable expression of E_5_ inclusion, all other patients showed differential expression of E_5_-included variants between paired T and N tissues. Particularly, the expression of S33/S39 without E_2_/E_3_ skipping was gained and lost in the T tissues of 6 (Ki#2, 9, Li#1, 3, 7 and Lu#3) and 4 (Ki#1, 4, 7, 8 and Lu#1) patients, respectively. Notably, nested PCR with E_2_F_1_/E_5_R_1_ showed that all 9 kidney cancer patients expressed E_5_ in their N tissues, of them 3 (Ki#1, 7, 8) lost E_5_ expression in their T tissues. By contrast, all 9 patients with liver cancer showed no E_5_ expression in their N tissues, and of them, 4 (Li#1, 2, 3, 7) gained E_5_ expression in their T tissues. The differential expression of E_5_-included variants between paired T and N tissues shown by nested PCR was supported by qRT-PCR assay of E_3_/E_5_ junction (Figure 4B).

In addition, the variant S18 which uses E_1c_ as the first exon was differentially expressed between paired T and N tissues for most (22/27) patients, with 12 and 10 patients showing increased and decreased expression, respectively (Figure 4B and 4C). Meanwhile, qRT-PCR assay indicated that the variants S1 and S11 which use E_1a_ and E_1b_ as the first exon, respectively, showed greater than 2-fold expression changes between paired T and N tissues for some patients (Figure 4B). Accordingly, alternative usage of the first exon (especially E_1c_) was differentially linked to different types of cancer.

### Regulation of E_3_ skipping by the selective PPARγ activator pioglitazone

**Figure 5:**
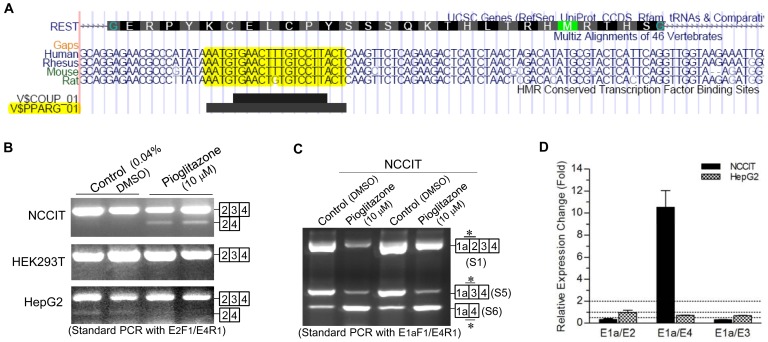
The effect of the selective PPARγ activator pioglitazone on *REST* E_3_ skipping. (**A**) Presence of an evolutionarily conserved PPARγ motif in *REST* E_3_. Bioinformatic data of the HMR Conserved Transcription Factor Binding Sites was retrieved from the UCSC Genome Browser (http://genome.ucsc.edu/cgi-bin/hgGateway). Cell-specific effect of pioglitazone (10 µM) on the expression of *REST* variants with/without E_3_ skipping was detected by standard PCR (**B** and **C**) and qRT-PCR (**D**). Standard PCR with E_2_F_1_/E_4_R_1_ showed that pioglitazone strikingly induced and slightly reduced E_3_ skipping in NCCIT and HepG2 cells, respectively, but exerted no effect in HEK293T cells. Regulation of E_3_ skipping by pioglitazone in NCCIT can also be observed by standard PCR with E_1a_F_1_/E_4_R_1_. Pioglitazone regulation of E_3_ skipping in NCCIT and HepG2 was further confirmed by qRT-PCR assay. Expression changes (pioglitazone over DMSO) were calculated by the 2^−ΔΔCt^ approach using *GAPDH* as the reference.

A view of the UCSC Genome Browser (http://genome.ucsc.edu/cgi-bin/hgGateway) indicates that E_3_ of *REST* contains a conserved binding motif for the peroxisome proliferator-activated receptor gamma (PPARγ) (Figure 5A), a ligand-activated nuclear receptor that contributes to cell differentiation and tumorigenesis besides its metabolic actions [40], [41]. Intriguingly, in searching for environmental factors that affect *REST* pre-mRNA splicing, we found that pioglitazone, a highly selective PPARγ agonist, exerts a cell-dependent effect on E_3_ skipping (Figure 5B-D). In NCCIT, a pluripotent stem cell line derived from human embryonic carcinoma, pioglitazone (10 µM) strikingly induced E_3_ skipping as indicated by increased expression of E_3_-skipped variants (S4 and S6) and decreased expression of E_3_-included variants (S1 and S5). In contrast, pioglitazone (10 µM) slightly reduced E_3_ skipping in HepG2 cells while it exerted no effect on E_3_ skipping in HEK293T cells.

## Discussion

**Figure 6:**
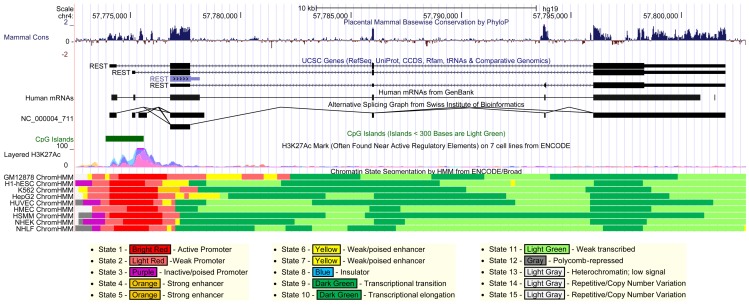
Bioinformatics at the *REST* gene locus. Data are retrieved from the UCSC Genome Browser (http://genome.ucsc.edu/cgi-bin/hgGateway). The *REST* promoter harbors a large-size CpG island and displays active transcription for all the 9 available cell lines without exception, as indicated by the chromatin state segmentation and histone H3 lysine 27 acetylation (H3K27Ac) tracks.

We reveal that *REST* undergoes extensive AS across an unexpectedly large gene boundary defined by a novel alternate last exon (E_5_), with numerous ncRNAs and coding mRNAs being expressed in a species- and cell-type/tissue-specific manner with individual differences. Notably, we found that exon (E_2_, E_3_ and E_4_) skipping is preferentially associated with the usage of the conventional E_1a_ as the first exon, i.e., AS of *REST* pre-mRNA is promoter-dependent, suggesting that alternative promoters differ not only in the strength, but also in transcription elongation which is a major determent of pre-mRNA splicing. Since most splice variants involve a complete or partial skipping of exons (e.g. E_2_, E_3_ and E_4_) encoding specific functional domains of REST, their functional significance is generally predictable. Accordingly, these alternatively spliced *REST* variants, which are mostly expressed in a cell-type/tissue-specific manner with individual differences, presumably contribute to the diverse, context-dependent regulation of *REST* gene expression. Notably, we found that all tissues and cell lines without exception express at least one *REST* splice variant, and no apparent difference in the expression pattern of specific splice variants was observed between neuronal and non-neuronal tissues. In agreement, similar levels of the initial *REST* transcripts were reported between neuronal and non-neuronal cells [42], while the promoter of *REST* exhibits cell-type-independent active transcription as indicated by publically available epigenetic data (Figure 6), suggesting that transcriptional regulation is unlikely a major contributor to cell-type/tissue-specific expression of REST. Hence, in line with epigenetic regulation of AS and the emerging role of AS in adaptive responses [2], [6], our findings strongly suggest that pre-mRNA splicing, rather than transcription regulation, substantially contributes to the diverse, context-dependent *REST* gene expression and function.

REST functions as a tumor suppressor or an oncogene depending on cellular context, with both increased and decreased REST activity having been reported in different types of cancer [18], [19]. In accordance, we found that numerous *REST* splice variants produced by E_2_/E_3_ skipping and alternative usage of the first and last exons, which are predictive of altered REST activity, are generally but differentially linked to different types of cancer. Particularly, in accordance with the involvement of REST4 and another C-terminal truncated REST-FS (caused by a frameshift mutation) in tumorigenesis [26]–[28], [43], we found that the usage of E_5_ as the last exon, which is predictive of a C-terminal truncated REST4-like protein, is differentially linked to various types of cancer. Unlike REST4 which exists in both primates and rodents, E_5_ inclusion is only expressed in human but it shows a wide tissue distribution, suggesting that it may contribute to the pathophysiology of a wide spectrum of human diseases. Besides encoding a REST4-like protein, E_5_-included variants overlap in opposite direction with *NOA1* transcript(s), i.e., transcripts of *REST* and *NOA1* act as natural antisense transcripts for each other, making it possible that transcripts of one gene regulate expression of the other gene through various mechanisms [44]. As a GTPase essential for mitochondrial protein synthesis, NOA1 is involved in oxidative stress and apoptosis [39], [45], [46]. Accordingly, E_5_ inclusion may mediate a coordinated effect of REST and NOA1 on cellular functions. In addition, E_5_ inclusion results in altered 3′-UTR, which contributes to posttranscriptional regulation (e.g. mRNA stability) of gene expression.

Since E_2_ skipping eliminates the TSS, E_2_-skipped variants may function as ncRNAs which potentially modulate *REST* gene expression. Similar to E_5_ inclusion which is exclusively expressed in human, E_2_ skipping is ubiquitously expressed in human but rarely expressed in nonhuman primates and rodents, suggesting that human evolution involves a gain of much more complex *REST* pre-mRNA splicing, which may contribute to context-dependent human genome function that is by far more complex than other species. As for the E_3_ skipping, it causes no frame shift but loss of ZFM-5 essential for nuclear translocation [32], [33], such that it provides an alternate mechanism for the regulation of REST activity. Notably, while REST-5FΔ caused by E_3_ skipping was only previously observed in SH-SY5Y cells [27], we revealed a ubiquitous though usually non-abundant expression of E_3_ skipping in primates, suggesting that E_3_ skipping might be a common regulator which specifies the diverse, context-dependent function of REST. In accordance, we found that numerous E_3_-skipped variants were differentially expressed between paired tumor and adjacent normal tissues for most patients with cancer, and that E_3_ skipping is modulated by the PPARγ activator pioglitazone which contributes to cell differentiation and tumorigenesis [40], [41], [47]. In addition, our preliminary data showed that E_3_ skipping is linked to virus-induced transformation of B-lymphocytes in both human and nonhuman primates (unpublished data). Hence, E_3_ skipping represents a potential biomarker for cancer. With respect to the alternative usage of the first exon, it alters the 5′-untranslated region which plays an important role in posttranscriptional regulation (e.g. mRNA stability, targeting and translation) of gene expression [37], such that it may affect the expression level of REST protein.

It has been documented that REST is a prognostic factor and therapeutic target for cancer [48], [49]. Accordingly, correction of aberrant *REST* pre-mRNA splicing provides a new strategy for the treatment of cancer. Indeed, pioglitazone regulation of E_3_ skipping strongly supports the feasibility of manipulating REST activity through modulation of pre-mRNA splicing. Since E_3_ contains a conserved binding motif for PPARγ, it is likely that the binding of activated PPARγ to E_3_ interrupts the action of other splicing factor(s) on E_3_ skipping/inclusion. To our knowledge, this is the first study reporting the regulation of pre-mRNA splicing by a ligand of PPARγ, which usually affects gene transcription [50]. PPARγ is implicated in a wide variety of biological processes including adipogenesis, glucose metabolism, inflammation and tumorigenesis, and it is the molecular target of the thiazolidinedione (TZD) class of antidiabetic drugs including pioglitazone and rosiglitazone [51]. It has been shown that TZDs suppress the growth of several cancer lines *in vitro* and *in vivo*, and lines of preclinical evidence supports the antineoplstic effects of PPARγ agonists; however, results from clinical trials show modest success [52]. Recently, the use of pioglitazone for type 2 diabetes mellitus is reportedly associated with an increased risk of bladder cancer [53], suggesting a context-dependent, bidirectional effect of PPARγ on tumorigenesis, which is in accordance with its cell-specific effect on E_3_ skipping (Fig.5), as well as the notion that both decreased and increased REST activity may contribute to tumorigenesis [18], [19]. Mechanism(s) by which PPARγ modulates tumorigenesis are not yet fully understood; however, based on the role of REST in tumorigenesis and the regulation of REST activity by E_3_ skipping, we speculate that PPARγ regulation of *REST* pre-mRNA splicing is attributable. Accordingly, the context-dependent REST activity modulated by E_3_ skipping may underlie the context-dependent effect of PPARγ on tumorigenesis, such that E_3_ skipping represents a potential therapeutic target that might be utilized for personalized medicine for cancer. Moreover, REST targets numerous genes involved in metabolism [54], suggesting that PPARγ regulation of *REST* pre-mRNA splicing may contribute, at least in part, to the metabolic actions of PPARγ. In this regard, this study provides a novel mechanism underlying the biological actions of PPARγ and the close link between metabolism and tumorigenesis [55]. Furthermore, PPARγ exerts a neuroprotective effect on neurodegenerative disorders including Huntington disease, which is caused by disassociation of mutant Huntingtin protein with REST in the cytoplasm and therefore enhanced nuclear translocation and aberrant accumulation of REST in nucleus [21], [56], [57]. It is tempting to speculate that the neuroprotective effect of PPARγ might be explained by its regulation of E_3_ skipping (presumably increased in neurons), which may result in reduced translocation of REST into the nucleus and alleviation of the repressive effect of REST on neuronal genes essential for the maintenance of neurons. Thus, E_3_ skipping may have implications for a wide variety of human diseases.

In summary, this study revealed an extensive AS of *REST* pre-mRNA and a close link between aberrant *REST* pre-mRNA splicing and various types of cancer. The findings not only advance our understanding of the complexity of *REST* gene expression and function, but also provide potential biomarkers and therapeutic targets for the diagnosis and individualized treatment of cancer. However, our findings require further validation in a large population of patients with different types and prognosis of cancer, and warrant further investigation of mechanisms underlying *REST* pre-mRNA splicing regulation and biological functions of specific *REST* splice variants.

## Supporting Information

Figure S1 **Detection of E_2_/E_3_ skipping and E_5_ inclusion in additional human cell lines and PBMCs.** Nested PCRs were performed by using the forward primers E_1a_F_1_ and E_2_F_1_ paired with the reverse primers E_4_R_1_ and E_5_R_1_, respectively.
(TIF)

Figure S2 **Individual difference in** ***REST*** **E_2_ skipping in rhesus monkeys.** The primer set E1aF2/E4R1 was employed to perform the standard PCR using cDNA samples from pons and raphe tissues from 4 rhesus monkeys. E_2_ skipping was observed in 1 of the 4 macaque pons and raphe tissues, respectively.
(TIF)

Figure S3 **Expression profile of** ***REST*** **splice variants in human cell lines.** The abundance of the expression was briefly estimated by the band of standard/nested PCRs and was color-encoded as indicated. The tissue distribution was given for variants (S9, S10, S14-S16, S24, S30, S36 and S41) that were not detected in cell lines. The glioblastoma tissue was obtained from UMass Cancer Center Tissue Bank. The GenBank accession numbers are given for 42 of the 45 variants.
(TIF)
