# Correction: Extensive Alternative Splicing of the Repressor Element Silencing Transcription Factor Linked to Cancer

**DOI:** 10.1371/annotation/330ce945-3e26-4314-bc57-708fb3c46af6

**Published:** 2014-01-17

**Authors:** Guo-Lin Chen, Gregory M. Miller

1. In Table 2, sequence of E2-F1 should be [5'-aagccatgccaatatgaagc-3']. It was given by mistake as the sequence of E2-R2, which can be paired with E2-F2 [sequence: 5'-ggaggaggagggctgtttac-3'] for qRT-PCR. The correct version of Table 2 can be viewed here: http://plosone.org/corrections/pone.0062217.t002.cn.tif

2. For nested PCR, if the 1st PCR product is not used for further quantification, a regular 35-40 cycle amplification for both 1st and 2nd PCRs is suggested to give more yield.

