# Supplementary figures and images for: Extensive Alternative Splicing of the Repressor Element Silencing Transcription Factor Linked to Cancer
Source: PLoS One. 2013 Apr 16;8(4):e62217. doi: 10.1371/journal.pone.0062217 (PMC3628349; doi:10.1371/journal.pone.0062217)

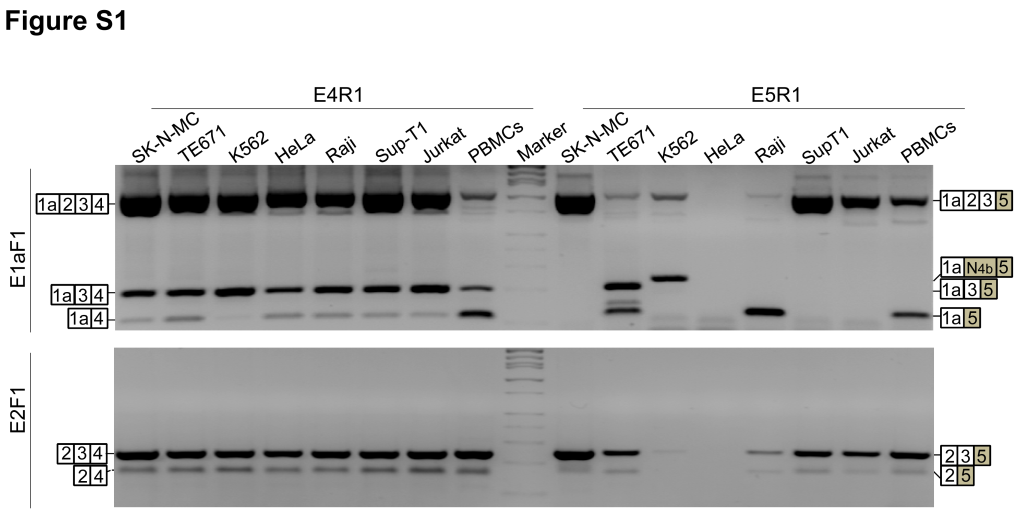

Supplement: Figure S1 — Detection of E2/E3 skipping and E5 inclusion in additional human cell lines and PBMCs. Nested PCRs were performed by using the forward primers E1aF1 and E2F1 paired with the reverse primers E4R1 and E5R1, respectively. (TIF) [file pone.0062217.s001.tif]

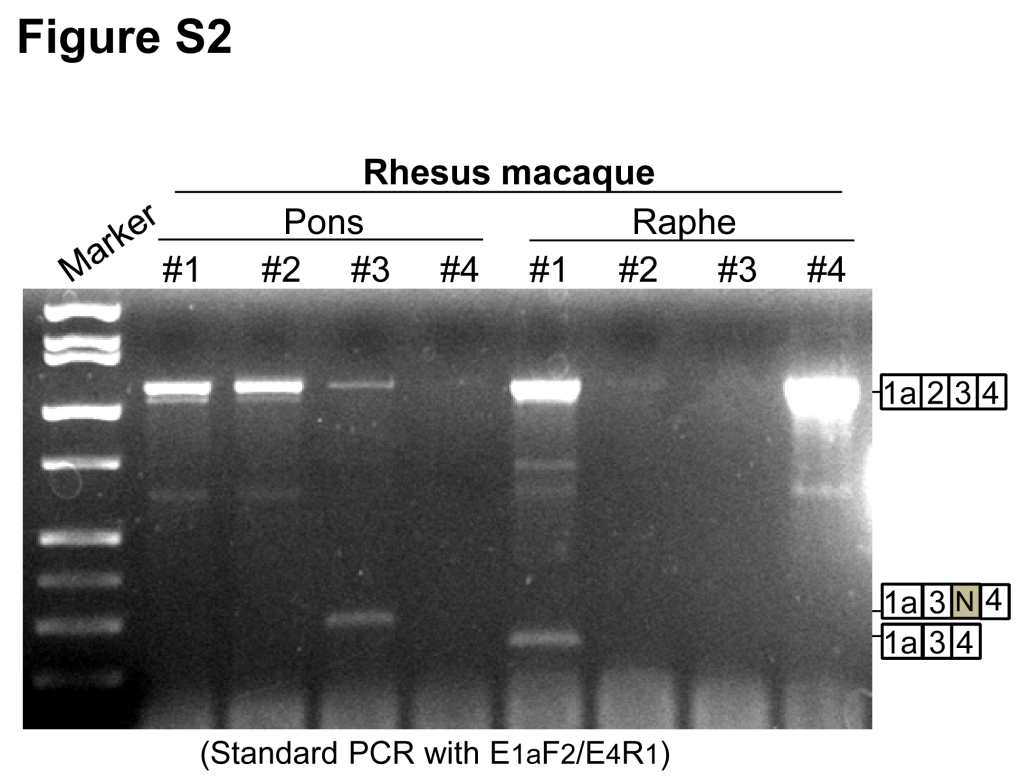

Supplement: Figure S2 — Individual difference in REST E2 skipping in rhesus monkeys. The primer set E1aF2/E4R1 was employed to perform the standard PCR using cDNA samples from pons and raphe tissues from 4 rhesus monkeys. E2 skipping was observed in 1 of the 4 macaque pons and raphe tissues, respectively. (TIF) [file pone.0062217.s002.tif]

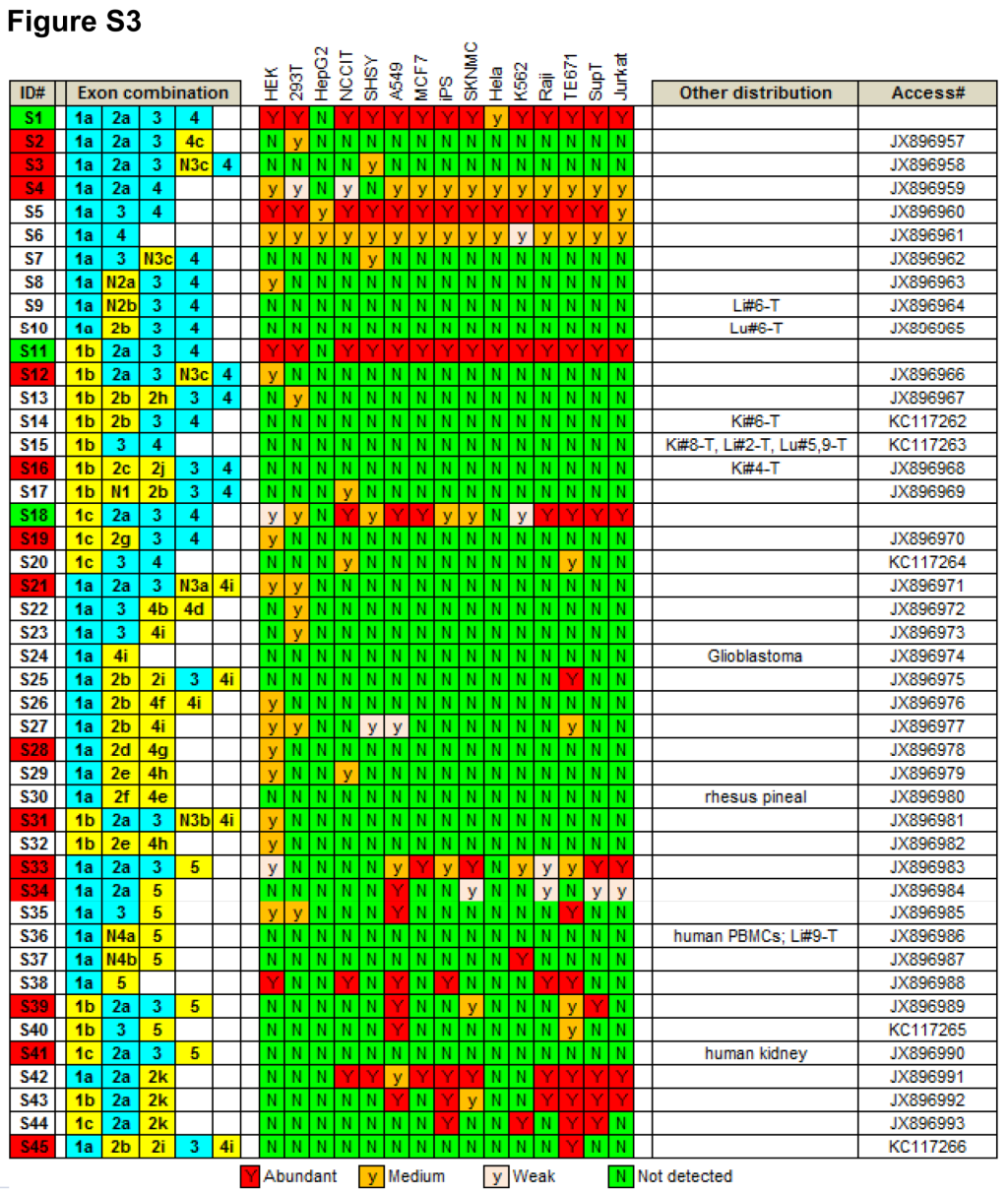

Supplement: Figure S3 — Expression profile of REST splice variants in human cell lines. The abundance of the expression was briefly estimated by the band of standard/nested PCRs and was color-encoded as indicated. The tissue distribution was given for variants (S9, S10, S14-S16, S24, S30, S36 and S41) that were not detected in cell lines. The glioblastoma tissue was obtained from UMass Cancer Center Tissue Bank. The GenBank accession numbers are given for 42 of the 45 variants. (TIF) [file pone.0062217.s003.tif]
